# Phyling: phylogenetic inference from annotated genomes

**DOI:** 10.1093/g3journal/jkag062

**Published:** 2026-03-26

**Authors:** Cheng-Hung Tsai, Jason E Stajich

**Affiliations:** Department of Microbiology & Plant Pathology, University of California-Riverside, Riverside, CA 92521, United States; Genetics, Genomics and Bioinformatics Graduate Program, University of California-Riverside, Riverside, CA 92521, United States; Department of Microbiology & Plant Pathology, University of California-Riverside, Riverside, CA 92521, United States; Institute for Integrative Genome Biology, University of California-Riverside, Riverside, CA 92521, United States

**Keywords:** phylogenetics, phylogenomics, software, orthology, hidden Markov models, python, bacteria, fungi, PEQG2026

## Abstract

Phyling is a fast, scalable, and user-friendly tool supporting phylogenomic reconstruction of species phylogenies directly from protein-encoded genomic data. It identifies orthologous genes by searching protein sequences against a curated set of hidden Markov model profiles, consisting of single-copy orthologs derived from the BUSCO database. To optimize the speed of the final inference, Phyling includes a module to filter aligned orthologs based on their phylogenetic informativeness. Finally, Phyling provides a companion wrapper for automated species tree construction using either consensus or concatenation strategies.

Phyling efficiently resolves large phylogenies by optimizing memory usage and data processing. Its checkpoint system enables users to incrementally add or remove samples without repeating the entire search process. For analyses involving closely related taxa, Phyling supports the use of nucleotide coding sequences, which may capture phylogenetic signals missed by protein sequences. The benchmark results show that Phyling substantially runs faster than OrthoFinder, a reciprocal best hit based method, while achieving equal or better accuracy.

## Teaser

Phyling reconstructs species phylogenies from protein-encoded sequences using BUSCO marker sets to identify orthologs. Benchmarks show Phyling runs significantly faster than OrthoFinder, a well-known phylogenetic inference tool, without sacrificing accuracy. Its streamlined interface makes it accessible to users with little phylogenetics background, while its flexible design supports advanced analyses such as concatenation-based inference and rapid adjustments via a checkpoint system.

## Introduction

Phylogenetic analysis provides the essential framework for deciphering evolutionary history and relationships among organisms (O’Meara 2012). Phylogenetic trees built from genetic data can trace the origin and evolution of genes, species, and traits; identify conserved biological functions; and reveal the processes driving species diversification and adaptation (Soltis and Soltis 2003; Yang and Rannala 2012).

Phylogenetic inference depends on models of sequence divergence, so choosing the right sequences is crucial to accuracy. Orthologous sequences, those that diverged through speciation from a common ancestor, are generally considered the most suitable markers for reconstructing species relationships (Kapli et al. 2020). However, phylogenetic studies are not strictly limited to orthologs and may incorporate other sequence types or phylogenetic approaches when necessary (Smith and Hahn 2021). A major strategy for identifying orthologs is similarity-based comparison, which clusters genes with high sequence similarity (Tekaia 2016; de Boissier and Habermann 2020). Two common implementations of this approach are reciprocal best hit (RBH) and profile-based matching.

RBH identifies orthologs by finding genes in two genomes that are each other's best match in bidirectional sequence similarity searches. In this approach, an all-against-all search is applied across samples, and clusters of RBH pairs are identified as orthologs. Several tools, such as OrthoMCL (Li et al. 2003), OrthoFinder (Emms and Kelly 2019), and OrthoPhy (Watanabe et al. 2023) adopt RBH combined with other clustering techniques like Markov Clustering to infer orthology.

In contrast, profile-based sequence matching identifies orthologs by clustering the sequences that match to the same profile in a database with known orthologous groups. Users match sequences in a genome against databases such as eggNOG (Hernández-Plaza et al. 2023), TreeFam (Ruan et al. 2008), and Pfam (Paysan-Lafosse et al. 2025), to classify the input sequences based on orthology or function. In addition to matching against general databases, some tools generate specialized datasets for bacteria or eukaryotes to further refine the inference (Lee 2019; Tice et al. 2021). Though conceptually distinct, RBH and profile-based matching are often used together. For example, eggNOG uses RBH for initial ortholog detection and profile hidden Markov models (HMMs) for efficient large-scale inference. This hybrid approach balances RBH's precision with the scalability of profile-based methods for better phylogenetic inference.

While all-against-all searches usually offer higher accuracy, they become computationally expensive as more genomes are added due to the quadratic growth in pairwise comparisons. Moreover, the rigid structure of the RBH-based pipelines makes it less flexible when updates are needed. Any change, such as adding or removing a single genome, requires repeating the entire search and clustering process. In contrast, profile-based approaches allow each sample to be processed independently, so users can incrementally update datasets without rerunning previously completed searches. This makes profile-based methods particularly convenient for large or frequently updated phylogenomic datasets.

Here we present Phyling, a fast and user-friendly tool for inferring phylogeny directly from genome data. Phyling utilized the profile-based ortholog identification strategy to achieve significant gains in speed and resource efficiency over RBH-based methods. Additionally, it can handle large-scale datasets of up to thousands of species, which are usually challenging for other tools. Phyling also features a checkpoint design that allows users to efficiently redo analyses without re-run searches on previously processed samples, further enhancing its scalability.

## Materials and methods

### Phyling pipeline

**Fig. 1.**
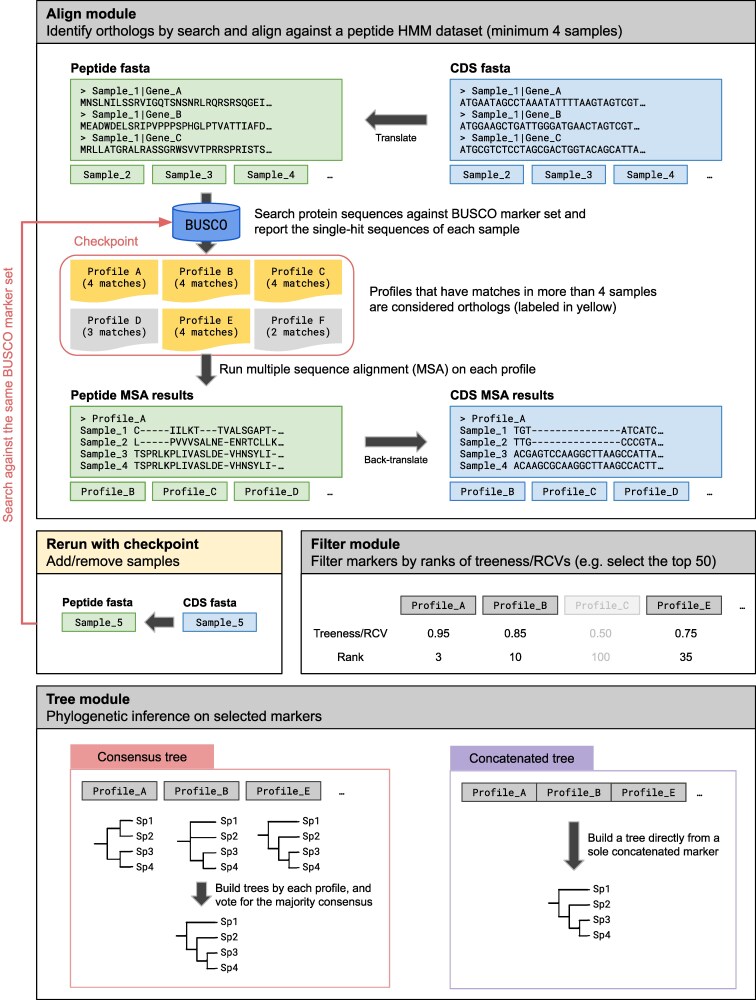
Flowchart of Phyling's core modules—align, filter, and tree. The download module is not shown. The Salmon-colored arrow indicates the checkpoint mechanism, in which hits from a newly added sample are incorporated into the existing hit collection from previous runs.

Phyling was mainly developed in Python version 3.9 and above and relies on a minimal set of command-line tools. To simplify installation and prevent dependency conflicts, we use Conda for managing the software environment and installing all required dependencies. The Python package Biopython version 1.81 and above (Cock et al. 2009) was used for sequence data processing. To allow more flexibility and user customization, Phyling is organized into four modules—download, align, filter, and tree (Fig. 1).

The download module facilitates, the retrieval of HMM profiles for the selected marker sets from the BUSCO database (Manni et al. 2021), which are used to identify orthologs in the input data. Upon first use, Phyling creates a directory under the user's home folder to store the downloaded marker sets.

The align module, the core of Phyling, identifies and aligns orthologous sequences across the input samples. Phyling requires a minimum of four samples and accepts either proteomes or nucleotide coding sequences. Ortholog identification is performed by searching sample sequences against predefined HMM profiles using hmmsearch from pyHMMER v0.11.0. (Larralde and Zeller 2023) To maximize the efficiency of pyHMMER's parallelization model, we implemented a dynamic CPU allocation wrapper. For analysis with less than eight CPUs assigned, a single instance utilizes all available cores. When equal or more than eight CPUs are assigned, the total pool is divided into nCPUs/4 parallel search instances, with each instance restricted to four cores. This strategy prevents the substantial performance decrease observed when a single pyHMMER instance is run with more than eight cores. The search parameters and hits are saved to a checkpoint file to enable reruns.

To ensure strict orthology, any sample exhibiting multiple hits to the same HMM profile is excluded for that marker. The orthologous sequences corresponding to the valid hits are then extracted using pyfaidx v0.8.1.3 (Shirley et al. 2015). By default, these sequences are aligned using hmmalign (from pyHMMER), although users have the option to switch to Muscle v5.3 (Edgar 2022) for potentially higher-quality alignments. Finally, the resulting alignments are trimmed by ClipKIT v2.1.1 (Steenwyk et al. 2020) to retain only the parsimony-informative sites for downstream phylogenetic analysis.

The filter module identifies and selects the most informative orthologs for downstream analysis. To quantify phylogenetic informativeness, a tree is first constructed for each aligned ortholog using FastTree v2.1.1 (Price et al. 2010). The resulting trees are then evaluated with the treeness over relative composition variability (RCV) score, computed by PhyKIT v2.0.1 (Steenwyk et al. 2021). Orthologs are ranked by their treeness/RCV scores, and the top *n* orthologs, defined by the user, are passed to the next stage of the workflow.

The tree module acts as an automated pipeline for phylogenetic inference, wrapping multiple tree building tools to process filtered orthologs. This eliminates the need for manual intervention between alignment and tree construction, facilitating a more efficient and reproducible workflow. Phyling offers two approaches: a species tree consensus (coalescence) approach or a concatenation (supermatrix) approach. By default, the workflow uses the consensus strategy, constructing individual gene trees with FastTree and inferring the final species tree with ASTER v1.19 (Zhang et al. 2018). For concatenation strategy, all alignments are combined, and ModelFinder (Kalyaanamoorthy et al. 2017) is used to select the best-fit model before tree inference with the user's choice of FastTree, RAxML-NG (Kozlov et al. 2019), or IQ-TREE (Minh et al. 2020). When using RAxML-NG or IQ-TREE, users may enable partitioned analysis to model locus heterogeneity more accurately (Chernomor et al. 2016). Branch support is assessed using both ultrafast bootstrap (UFBoot; (Hoang et al. 2018)) and the site concordance factor (Mo et al. 2023).

### Dataset for benchmark

To demonstrate the performance of Phyling, we utilized three distinct data sources: the Ensembl Genomes (Release 60) collection (Yates et al. 2022), a broad eukaryotic phylogeny dataset, and a simulated dataset for accuracy validation. For the Ensembl-based benchmarks, we retrieved metadata for 31,332 bacterial and 1,505 fungal strains, which were subset into specific benchmarking scenarios.

We categorized the Ensembl subsets into two types to evaluate Phyling across varying phylogenetic depths. The “general dataset” represents common research applications focused on family-level resolution. The fungal general dataset consists of 30 samples, including 28 from the family Saccharomycetaceae and two outgroups from Phaffomycetaceae. Correspondingly, the bacterial general dataset includes 96 samples—94 from Staphylococcaceae and two Bacillaceae outgroups.

To simulate more challenging conditions involving greater evolutionary divergence, we constructed “distant datasets” by selecting one representative strain per family and per order from the full Ensembl collection. This selection resulted in 251 bacterial and 231 fungal samples, providing a rigorous test for the tool's ability to resolve deeper nodes. Detailed metadata for the Ensembl datasets are provided in Supplementary File 1.

To assess the limitations of Phyling regarding extremely broad phylogenies, we assembled a broad eukaryotic phylogeny dataset of 165 diverse eukaryotic proteomes. This was curated by selecting one representative genus from the UniProt collection (UniProt Consortium 2025) for each genus represented in the EukPhylo study (Katz et al. 2025), which served as a comprehensive comparative framework for our reconstruction. Notably, all Microsporidia were excluded from this analysis due to their highly reduced genomes, which often lack the conserved marker density required for broad-scale orthology assessment. Detailed metadata for the resulting 165 samples are also provided in Supplementary File 1.

Finally, to establish a “ground-truth” for inference accuracy, we utilized a simulated dataset of 96 *Streptococcus pneumoniae* strains (Lees et al. 2018). This dataset was artificially evolved from the *S. pneumoniae* ATCC 700669 reference genome using a defined phylogenetic framework originally inferred by Kremer et al. (2017). Because the evolutionary history of these sequences is known, this dataset allows for a precise quantitative assessment of Phyling's topological accuracy.

### Benchmark

To monitor computational resource usage, including time and memory consumption, we employed the Python package memory-profiler v0.61.0 (Pedregosa 2022) during all benchmarks. Each tool was executed three times under identical conditions on a high-performance computing cluster equipped with AMD EPYC 7713 64-core processors (base clock 2 GHz, boost clock up to 3.67 GHz) to ensure consistency and reproducibility of the performance measurements.

Generalized Robinson–Foulds (RF) distance implemented in R library TreeDist (Smith 2020) was used to measure distances between trees inferred by different workflows. Non-metric multidimensional scaling (NMDS) was performed on RF distance matrices using the Python package scikit-learn (Pedregosa et al. 2011) for visualization. The R library MonoPhy (Schwery and O’Meara 2016) was used to assess the monophyly status of tree pairs constructed by different workflows, as shown in Supplementary Table 3 and Supplementary Fig. 3.

### Data visualization

We used the Python package matplotlib (Hunter 2007) and its high-level interface seaborn (Waskom 2021) to visualize most of the results. R libraries ggplot2 (Wickham 2009) and ggtree (Yu et al. 2017) were used for phylogenetic tree visualization.

## Results

### Phyling is fast and memory efficient

**Fig. 2.**
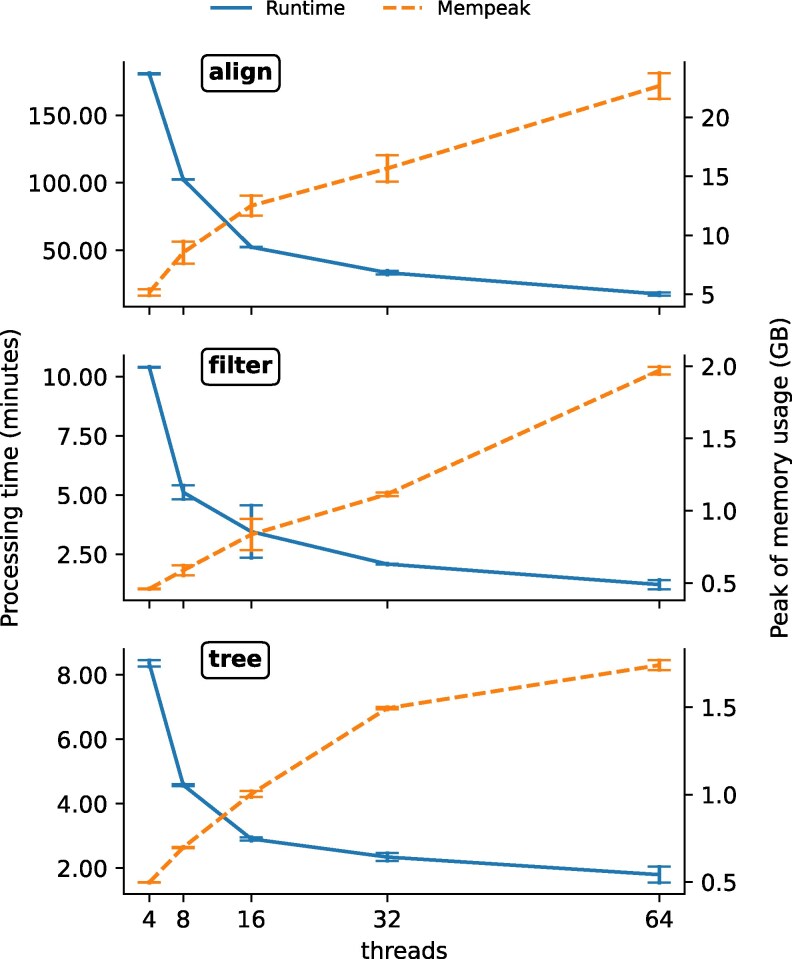
The multithreading performance benchmark for each module of Phyling. Blue solid lines (left *y*-axis) represent runtime in minutes and orange dashed lines (right *y*-axis) indicate peak memory usage in GB. Error bars represent standard deviations across three trials.

The optimized parallelization and data processing design contributes to the speed of Phyling and allows it to perform analysis on over thousands of samples. Overall, Phyling runs most efficiently with 16 threads when benchmarking with the fungal distant dataset and the acceleration has limited improvement when applying more than 32 threads (Fig. 2). Therefore, 16-thread configuration will be used in the following comparison benchmarks.

**Fig. 3.**
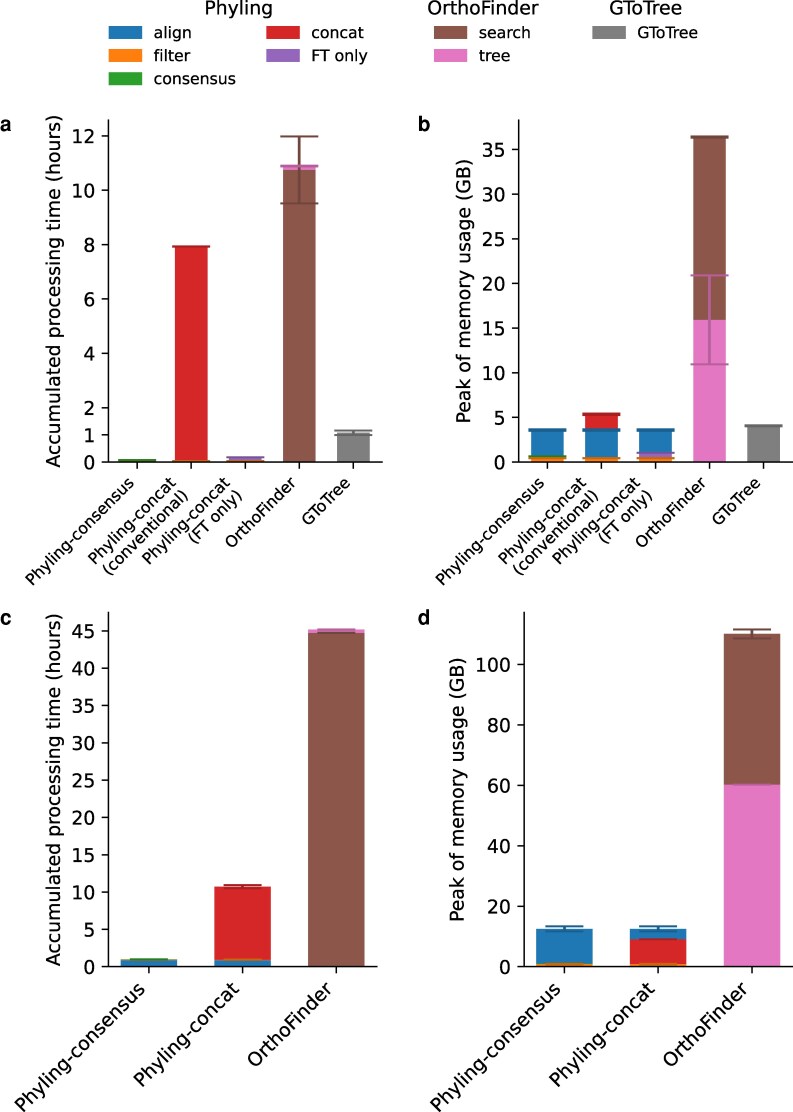
Accumulated runtime and peak memory usage for different modes of Phyling, OrthoFinder, and GToTree on distant datasets. (a, b) Bacterial dataset; (c, d) fungal dataset. Left panels (a, c) show accumulated runtime, and right panels (b, d) show peak memory usage. The conventional concatenation mode of Phyling performs best-fit model selection, followed by tree inference with FastTree, and branch support assessment as described in Materials and Methods section. The alternative “FT only” concatenation mode runs FastTree with LG model and discrete gamma distribution directly on the concatenated sequence made by the conventional pipeline. Colors represent different tools and modules, and error bars represent standard deviations across three trials.

To benchmark the performance of Phyling, we compared it with OrthoFinder, one of the widely used phylogenetic inference tools based on RBH, and GToTree (Lee 2019), a tool which is also developed upon profile-based approach to demonstrate the performance. When benchmarking the bacterial distant dataset with BUSCO bacteria_odb10 marker set (contains 124 markers), Phyling took less than 5 min on average with consensus mode, faster than both OrthoFinder and GToTree (using Bacteria SCG-set which contains 74 profiles) that took approximately 11 and 1 h, respectively (Fig. 3a and Supplementary Table 1). In contrast, the more comprehensive concatenation mode took over 8 h which is still faster than OrthoFinder. The UFBoot accounted for a large portion of the time spent in concatenation mode. By default, GToTree runs FastTree to infer phylogenies from concatenated sequences. To enable a more direct comparison, we used the concatenated sequences generated by Phyling's tree module and ran FastTree independently. With this alternative workflow, Phyling completed the analysis in approximately 10 min, faster than GToTree, which required nearly an hour. Phyling's peak memory usage was only about 10% to 15% of that of OrthoFinder in the bacterial dataset depending on the inference mode (Fig. 3b and Supplementary Table 2). Both Phyling and GToTree are very memory efficient tools, using at most 5 GB during the run.

When benchmarking the fungal distant dataset with BUSCO fungi_odb10 marker set (contains 758 markers), Phyling completed the analysis in less than 1 h on average using the consensus mode, and approximately 10 h with concatenation mode (Fig. 3c and Supplementary Table 1). Both modes were substantially faster than OrthoFinder, which took around 45 h to complete. The major factor contributing to the difference in processing time is the number of orthologs used for phylogenetic inference. OrthoFinder uses 44,092 orthogroups in its analysis, whereas Phyling utilizes only 754. We do not include the GToTree results since there is no suitable marker set for fungi inference. The only relevant marker set, Universal-Hug-et-al (Hug et al. 2016), contains only 16 markers, and most of the genomes were removed during the filtering process. In terms of memory usage, Phyling's peak memory consumption was only about 10% of that observed for OrthoFinder (Fig. 3d and Supplementary Table 2).

### Phyling achieves equal or better accuracy in species tree inference compared to OrthoFinder

**Fig. 4.**
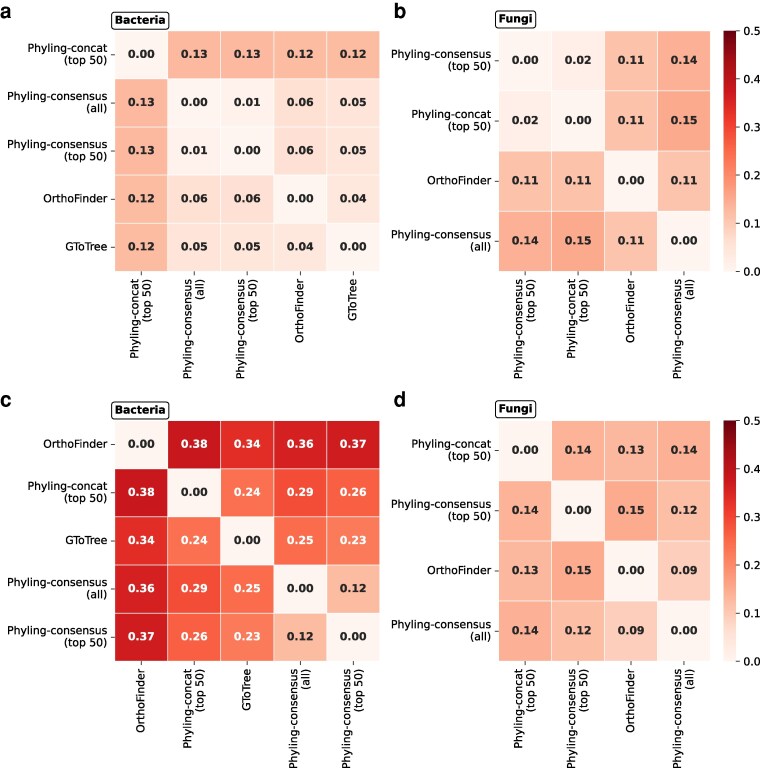
Generalized RF distance matrices among trees inferred by different tools. (a, b) General datasets; (c, d) distant datasets. Left panels (a, c) show bacterial results, and right panels (b, d) show fungal results. Matrices are hierarchically clustered, with color scales truncated to 0 to 0.5 to highlight variation.

To assess the accuracy of Phyling's phylogenetic inference, we computed the generalized RF distance between its trees and those constructed by OrthoFinder and GToTree across both the general and distant datasets. Overall, the inferences made by Phyling, under both consensus and concatenation modes, are highly congruent with those made by OrthoFinder and GToTree, in both bacterial and fungal general datasets (Fig. 4a and 4b). When evaluated using the fungal distant dataset, trees generated by Phyling also exhibited a high degree of topological similarity to those produced by OrthoFinder (Fig. 4c and 4d). This similarity between the trees was higher when all available orthologs were used rather than using a filtered subset restricted to the top 50 scoring orthologs. Benchmarking with the bacterial distant dataset revealed greater topological divergence among the three workflows, suggesting higher complexity or variability in bacterial phylogenetic signals. When using phylum-level taxonomy to assess the monophyly of resulting trees, all three methods performed comparably with GToTree having a slightly higher number of monophyletic taxa (Supplementary Fig. 1).

To evaluate Phyling's performance on highly divergent datasets, we analyzed a broad eukaryotic phylogeny using the eukaryota_odb12 marker set (contains 129 markers). Phyling successfully assigned the most of taxa to their respective kingdom- and phylum-level groups (Supplementary Fig. 2). However, we observed some topological instability within the SAR and Metamonada clades. This is likely due to a fewer available markers for these groups compared to the more robustly represented Viridiplantae and Opisthokonta (Supplementary File 1). Overall, these results demonstrate that with an appropriately selected marker set, Phyling is capable of resolving complex, large-scale eukaryotic relationships.

**Fig. 5.**
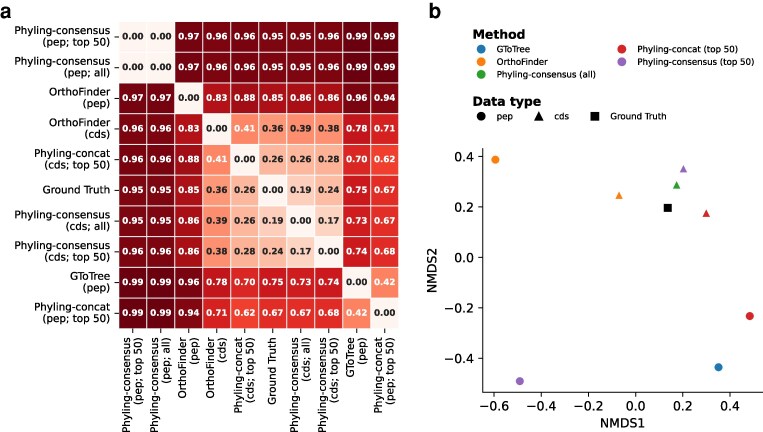
Distance among trees inferred by different tools using the simulated dataset. a) Generalized RF distance matrix, hierarchically clustered with values ranging from 0 to 1. b) Corresponding NMDS plot. Colors represent inference methods, shapes denote data types, and the black square indicates the ground-truth tree. Nucleotide-based inference with GToTree was not performed, as this option is available only when working with unannotated genomes.

To further assess Phyling's performance on datasets comprising close lineages, we evaluated it using a simulated dataset with known phylogenies described by Lees et al. Across all three workflows, phylogenetic inferences based on peptide sequences were generally far less accurate than those derived from coding nucleotide sequences (Fig. 5). Among the workflows, the Phyling tree had the highest topological similarity to the reference tree when using coding nucleotide sequences, particularly when inferring by the concatenation mode. The accuracy slightly improved when using the streptococcaceae_odb12 marker set (contains 689 markers) under both the concatenation mode and the consensus mode utilizing all orthologs, compared to the more general bacteria_odb10 marker set. However, the phylogenetic tree inferred using only the top 50 orthologs from the streptococcaceae_odb12 set showed the lowest accuracy among all tested combinations of marker sets and inference modes (Supplementary Fig. 3). This result highlights that employing a taxon-specific marker set does not consistently guarantee optimal performance, particularly when using a reduced subset of orthologs.

## Discussion

Phylogenetic analysis is a crucial step in comparative and population genomics, and Phyling streamlines this process with speed and accuracy. Taking advantage of the extensive ortholog collections from BUSCO, users can easily select an appropriate marker set for their analyses. Even with a more generalized marker set, Phyling still can offer relatively accurate results (Fig. 5). By employing HMM-based profile matching and optimized parallelization strategy through PyHMMER, Phyling processes large datasets significantly faster than RBH-based methods and even traditional HMMER searches. With all these user-friendly setups and processing speed, Phyling does not sacrifice the accuracy of the inference.

An additional advantage of using the BUSCO marker set for ortholog identification is the reduction of errors in phylogenetic inference caused by gene duplication events. Since phylogenetic reconstruction fundamentally depends on accurately identified orthologs, misidentification can lead to incorrect tree topologies (Tekaia 2016; Emms and Kelly 2019; de Boissier and Habermann 2020; Kapli et al. 2020; Steenwyk et al. 2023). The BUSCO marker sets are composed of genes that are expected to be present in single copy across a wide taxonomic range, thereby minimizing the inclusion of paralogs. Furthermore, Phyling incorporates an additional filtering step, similar to that implemented in GToTree (Lee 2019), to exclude sequences from a sample if multiple hits are found for the same marker to further enhance orthology.

The concatenation mode offers more dedicated inference and additional branch support information than speed-focus consensus mode. In addition to bootstrap values, Phyling incorporates site concordance factors from IQ-TREE to provide users with complementary insights into gene tree discordance. This is particularly valuable given that topological variation is not always captured by bootstrap values, especially when inferencing from a concatenated dataset (Mendes and Hahn 2018; Lanfear and Hahn 2024). However, these steps increase runtime. Automatic model selection and bootstrapping are the main contributors, as both involve iterative optimization. In particular, UFBoot may take longer to converge when samples are highly phylogenetically divergent.

Profile-based methods offer not only speed but also significant advantages in memory efficiency. In RBH-based approaches, identifying orthogroups requires exhaustive pairwise comparisons across all samples, which can create a bottleneck on systems with limited memory due to the accumulation of intermediate files. In contrast, the profile-based methods directly identify best hits against a predefined marker set without the need for pairwise comparisons, resulting in much lower memory usage. This efficiency enables Phyling to scale effectively to datasets with thousands of samples.

A primary concern is whether a small subset of orthologs—approximately two orders of magnitude fewer than those identified by tools like OrthoFinder in our benchmark with the fungal dataset—is sufficient for accurate phylogenetic reconstruction. While including more orthologs can improve accuracy (Rokas and Carroll 2005; Secaira-Morocho et al. 2025), the quality of the orthologs (e.g. those that are single-copy and widely shared) is often more paramount than mere quantity (Walden and Schranz 2023; Alam et al. 2025). Orthologs filtering becomes essential when genes exhibit high topological heterogeneity due to incomplete lineage sorting, which can drive discordance between individual gene trees and the underlying species phylogeny (Mendes and Hahn 2016; Molloy and Warnow 2018). Consequently, a smaller subset of high-quality orthologs is often sufficient to produce robust phylogenetic inference (Rokas et al. 2003; Ke et al. 2017; Li et al. 2017). It should be noted, however, that aggressive filtering may introduce ascertainment bias (Lewis 2001), potentially overestimating branch lengths if the resulting ortholog subset contains insufficient invariant sites. In such instances, a more inclusive ortholog set is advisable to maintain branch length accuracy.

Building on the characteristic of profile-based methods, Phyling incorporates a checkpoint mechanism in its align module. This feature allows users to adjust the sample set for phylogenetic analysis while reusing previous search results. When a search is completed, the results are saved to a checkpoint file, which can be loaded during a rerun. This ensures that only newly added or modified samples are searched, greatly reducing computation time for large datasets. It is important to note that the checkpoint functionality is limited to the search step, as the outputs it generates differ fundamentally and cannot be reused in subsequent steps.

Beyond reruns, the checkpoint system in Phyling also serves as a powerful feature for extending analyses. Hits are associated with samples using a hash of their file content, rather than relying on file names or paths. This content-based identification makes checkpoint files portable and shareable. Along with the original sequencing files, an existing checkpoint can serve as a prebuilt backbone to match new samples. This approach enables efficient integration of additional samples into established phylogenies, making it especially useful for identifying and placing newly sequenced or uncharacterized species within a broader evolutionary framework.

In summary, Phyling offers a robust, efficient, and scalable solution for phylogenetic inference in comparative and population genomics. Its ability to integrate well-established marker sets, handle large and evolving datasets, and provide meaningful support metrics makes it a versatile tool for phylogenomic studies. Additionally, the simplified interface lowers the barrier for less experienced users to perform phylogenetic analyses, while advanced users retain the flexibility to customize their workflows using intermediate outputs.

## Supplementary Material

jkag062_Supplementary_Data

## Data Availability

The Phyling package is available at https://github.com/stajichlab/PHYling and Zenodo doi: https://doi.org/10.5281/zenodo.1257001. The analyses described in this study were performed using the release v2.3.0 archived as doi: https://doi.org/10.5281/zenodo.16415735. The preprint of this work is available on bioRxiv at https://doi.org/10.1101/2025.07.30.666921. The genomic data and metadata utilized in this study are derived from publicly available repositories. Supplementary File 1 provides a comprehensive list of all taxonomic information, accession numbers, and direct download links for the Ensembl and broad eukaryotic phylogeny datasets. The simulated ground-truth dataset was obtained from the study by Lees et al. and is hosted at https://dx.doi.org/10.6084/m9.figshare.5483461. The amino acid (“aa”) and nucleotide (“dna”) FASTA files located within the “DB” folder were utilized as input sequences, while the “RealTree.nwk” file served as the reference topology (ground truth) for accuracy assessment. Supplemental material is available at G3 online.
