# Supplementary material for: Phyling: phylogenetic inference from annotated genomes
Source: G3 (Bethesda). 2026 Mar 26;16(5):jkag062. doi: 10.1093/g3journal/jkag062 (PMC13148406; doi:10.1093/g3journal/jkag062)
Supplement: jkag062_Supplementary_Data [file jkag062_supplementary_data.zip › Supplementary_Tables_and_Figures_G3-2025-406414.pdf]

Supplementary Tables

Supplementary Table 1. Runtime of different tools and modules.

Tool	Module	Dataset	Mean (min)	Standard deviation	Standard error of mean
Phyling	align	bacterial distant	1.95	0.01	0.00
Phyling	filter	bacterial distant	0.32	0.00	0.00
Phyling	consensus	bacterial distant	2.24	0.01	0.00
Phyling	concat	bacterial distant	473.44	0.29	0.17
Phyling	FT only	bacterial distant	8.08	0.02	0.01
OrthoFinder	search	bacterial distant	644.93	73.81	42.61
OrthoFinder	tree	bacterial distant	8.52	1.04	0.60
GToTree	GToTree	bacterial distant	64.77	5.08	2.93
Phyling	align	fungal distant	52.36	0.14	0.08
Phyling	filter	fungal distant	3.46	1.11	0.64
Phyling	consensus	fungal distant	2.91	0.05	0.03
Phyling	concat	fungal distant	586.42	13.38	7.73
OrthoFinder	search	fungal distant	2,684.34	1.07	0.62
OrthoFinder	tree	fungal distant	26.19	0.82	0.47

Mean, Standard deviation and Standard error of mean are calculated across three trials

Supplementary Table 2. Peak memory usage of different tools and modules.

Tool	Module	Dataset	Mean (GB)	Standard deviation	Standard error of mean
Phyling	align	bacterial distant	3.58	0.08	0.05
Phyling	filter	bacterial distant	0.44	0.01	0.00
Phyling	consensus	bacterial distant	0.64	0.00	0.00
Phyling	concat	bacterial distant	5.35	0.08	0.04
Phyling	FT only	bacterial distant	1.04	0.00	0.00
OrthoFinder	search	bacterial distant	36.39	0.06	0.04
OrthoFinder	tree	bacterial distant	15.92	4.98	2.87
GToTree	GToTree	bacterial distant	4.06	0.05	0.03
Phyling	align	fungal distant	12.51	0.84	0.49
Phyling	filter	fungal distant	0.84	0.11	0.06
Phyling	consensus	fungal distant	1.00	0.02	0.01
Phyling	concat	fungal distant	9.04	0.02	0.01
OrthoFinder	search	fungal distant	110.08	1.49	0.86
OrthoFinder	tree	fungal distant	60.22	0.01	0.01

Mean, Standard deviation and Standard error of mean are calculated across three trials

Supplementary Table 3. Monophyly test summary of phylogenetic inference made by different tools using the bacterial distant dataset.

	Phyling		GToTree		OrthoFinder	
	#Taxa	#Tips	#Taxa	#Tips	#Taxa	#Tips
Total	50	251	50	251	50	251
Monophyletic	18	171	20	176	18	144
Non-Monophyletic	7	54	5	49	7	81
Monotypic	25	25	25	25	25	25
Intruder	3	4	4	5	17	32
Outlier	4	4	2	2	3	4

#Taxa: number of phylum-level taxa assigned to each category

#Tips: column represents the total number of tips falling into each category

Monotypic: taxa represented by only a single tip

Intruder: clades that appear within another taxon's clades

Outliers: single-tip intruders which are too far away from the rest of their taxon

Supplementary Figures

A

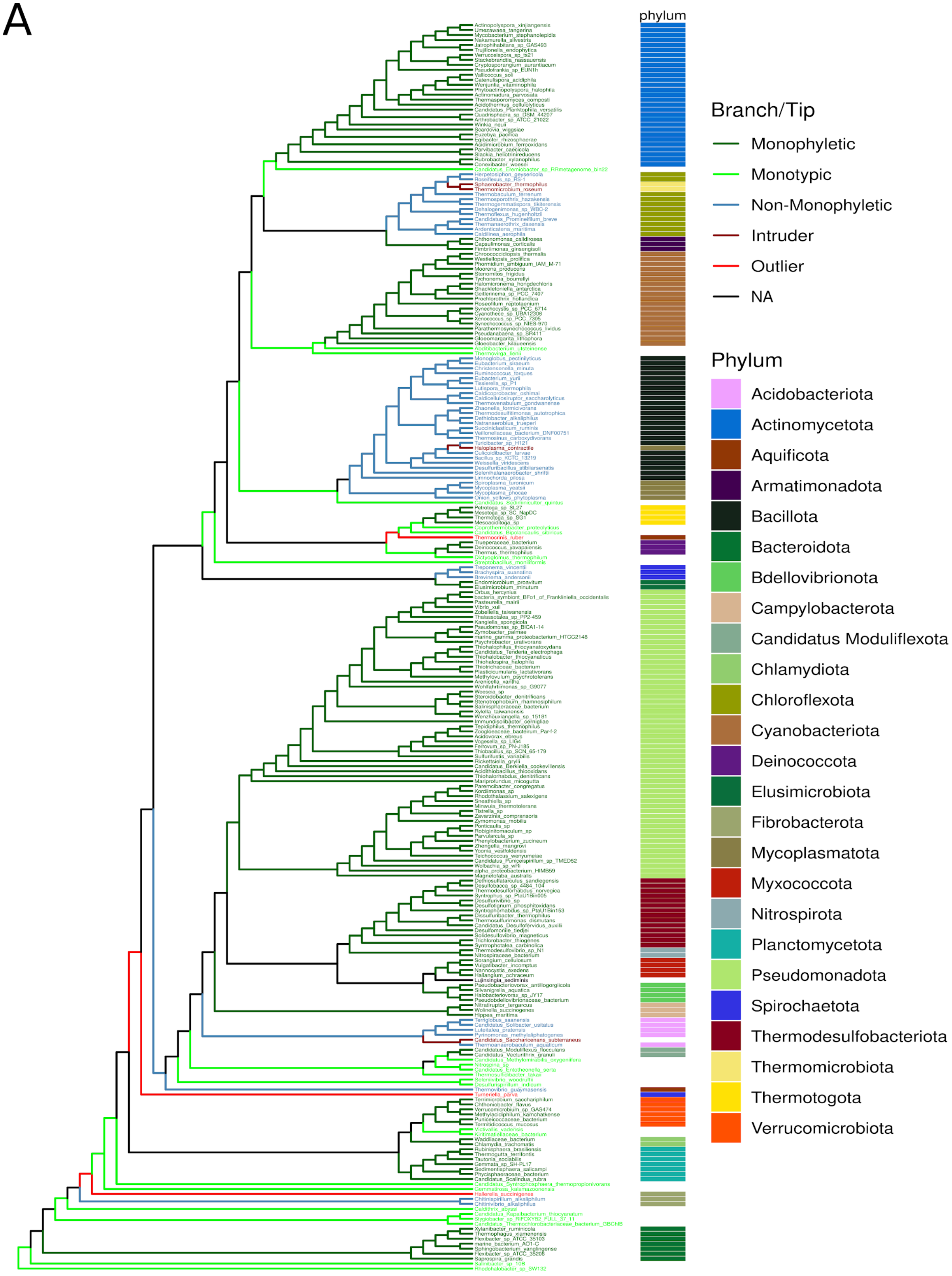

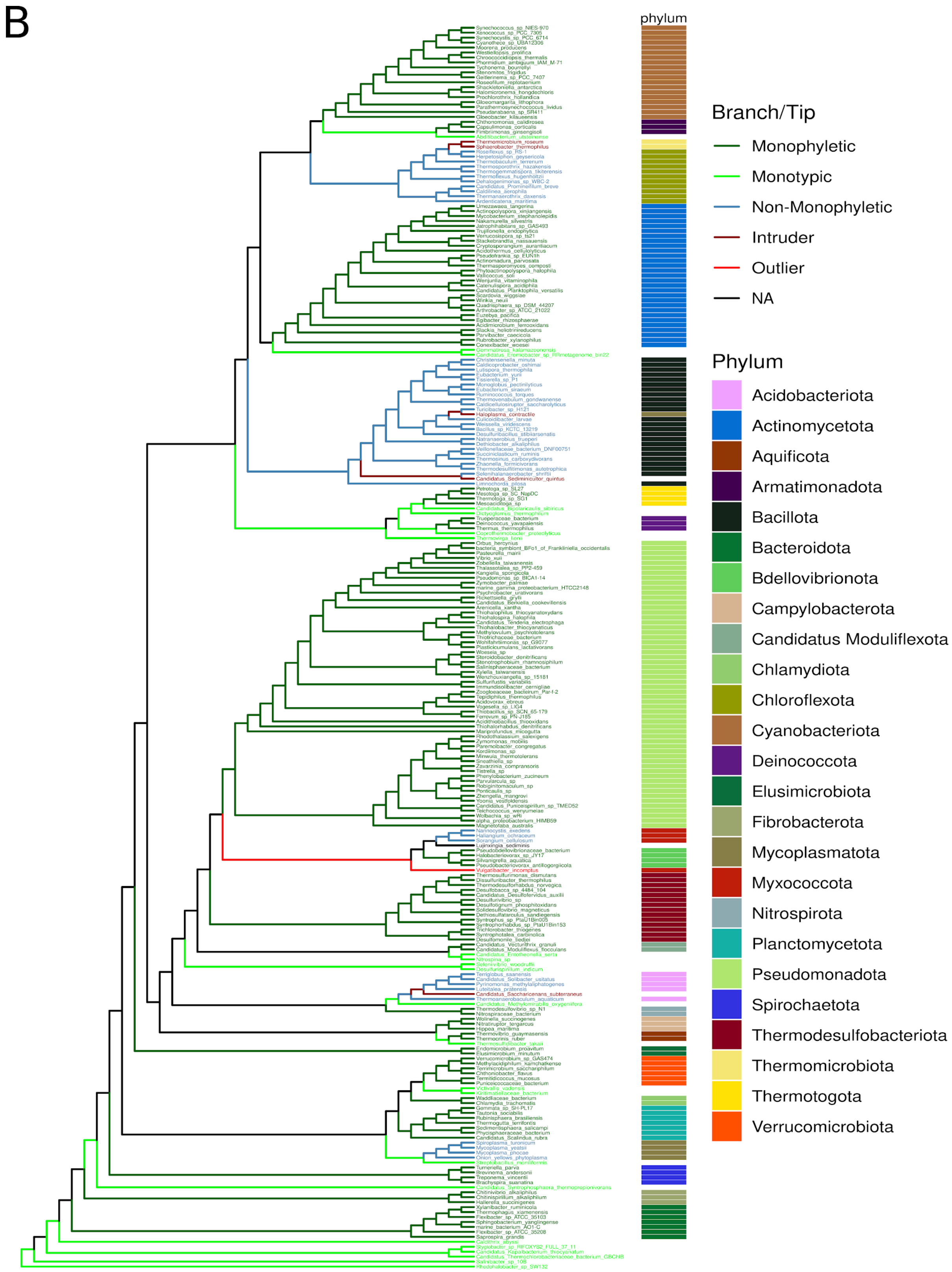
B
phylum
Branch/Tip
Monophyletic
Monotypic
Non-Monophyletic
Intruder
Outlier
NA
Phylum
Acidobacteriota
Actinomycetota
Aquificota
Armatimonadota
Bacillota
Bacteroidota
Bdellovibrionota
Campylobacterota
Candidatus Moduliflexota
Chlamydiota
Chloroflexota
Cyanobacteriota
Deinococcota
Elusimicrobiota
Fibrobacterota
Mycoplasmatota
Myxococcota
Nitrospirota
Planctomycetota
Pseudomonadota
Spirochaetota
Thermodesulfobacteriota
Thermomicrobiota
Thermotogota
Verrucomicrobiota

C

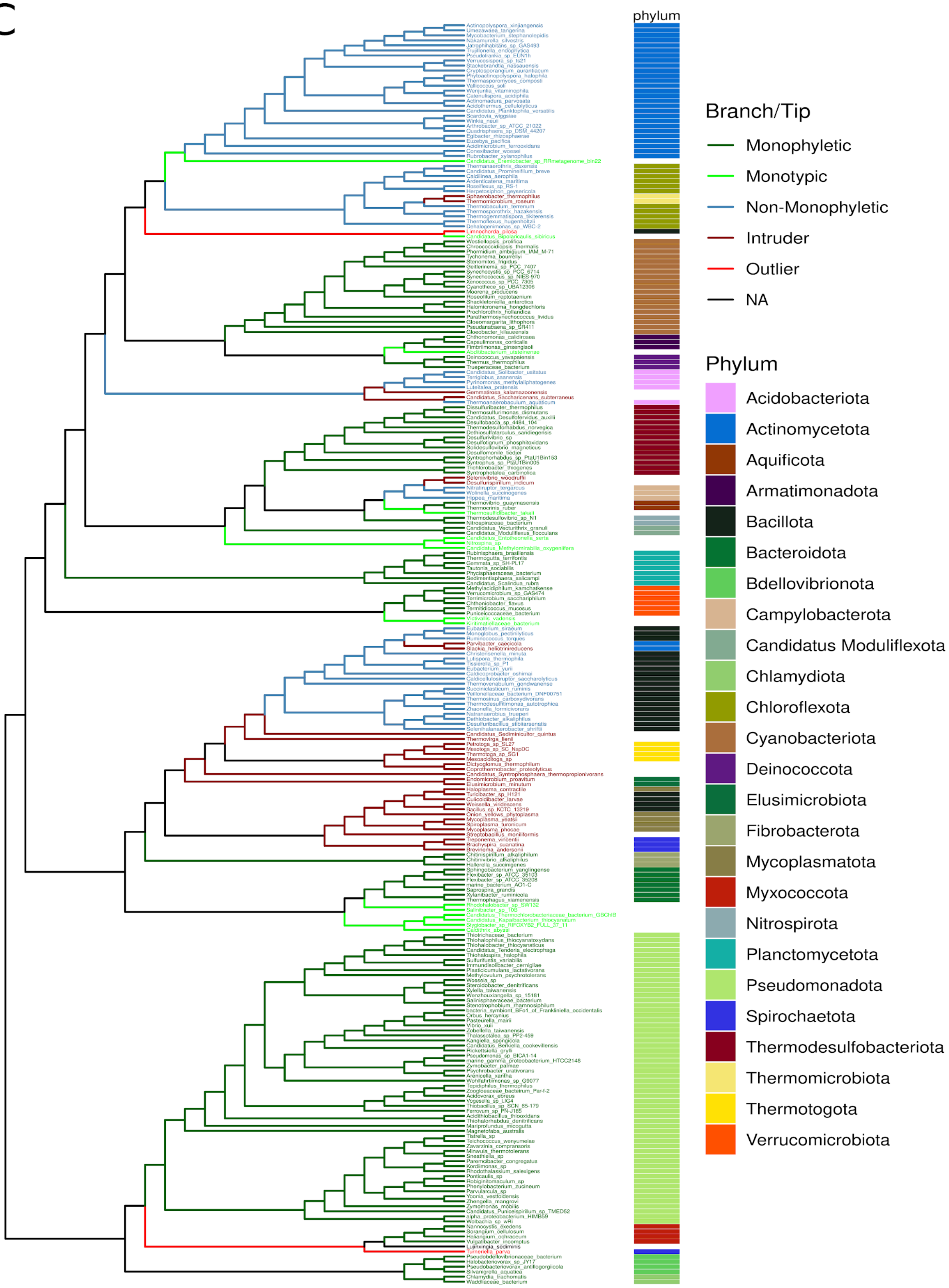
phylum
Branch/Tip
Monophyletic
Monotypic
Non-Monophyletic
Intruder
Outlier
NA
Phylum
Acidobacteriota
Actinomycetota
Aquificota
Armatimonadota
Bacillota
Bacteroidota
Bdellovibrionota
Campylobacterota
Candidatus Moduliflexota
Chlamydiota
Chloroflexota
Cyanobacteriota
Deinococcota
Elusimicrobiota
Fibrobacterota
Mycoplasmatota
Myxococcota
Nitrospirota
Planctomycetota
Pseudomonadota
Spirochaetota
Thermodesulfobacteriota
Thermomicrobiota
Thermotogota
Verrucomicrobiota

Supplementary Figure 1. Cladogram of the phylogenetic inference made by different tools using the bacterial distant dataset. (A) Phyling (consensus mode); (B) GToTree; and (C) OrthoFinder. The branch and tip colors indicate monophyly status, where the blue branches, dark red and red highlight non-monophyletic clades and their corresponding intruding tips, indicate potential misclassifications. Color labels on the right represent the phylum-level taxonomic assignments for each tip, excluding monotypic tips.

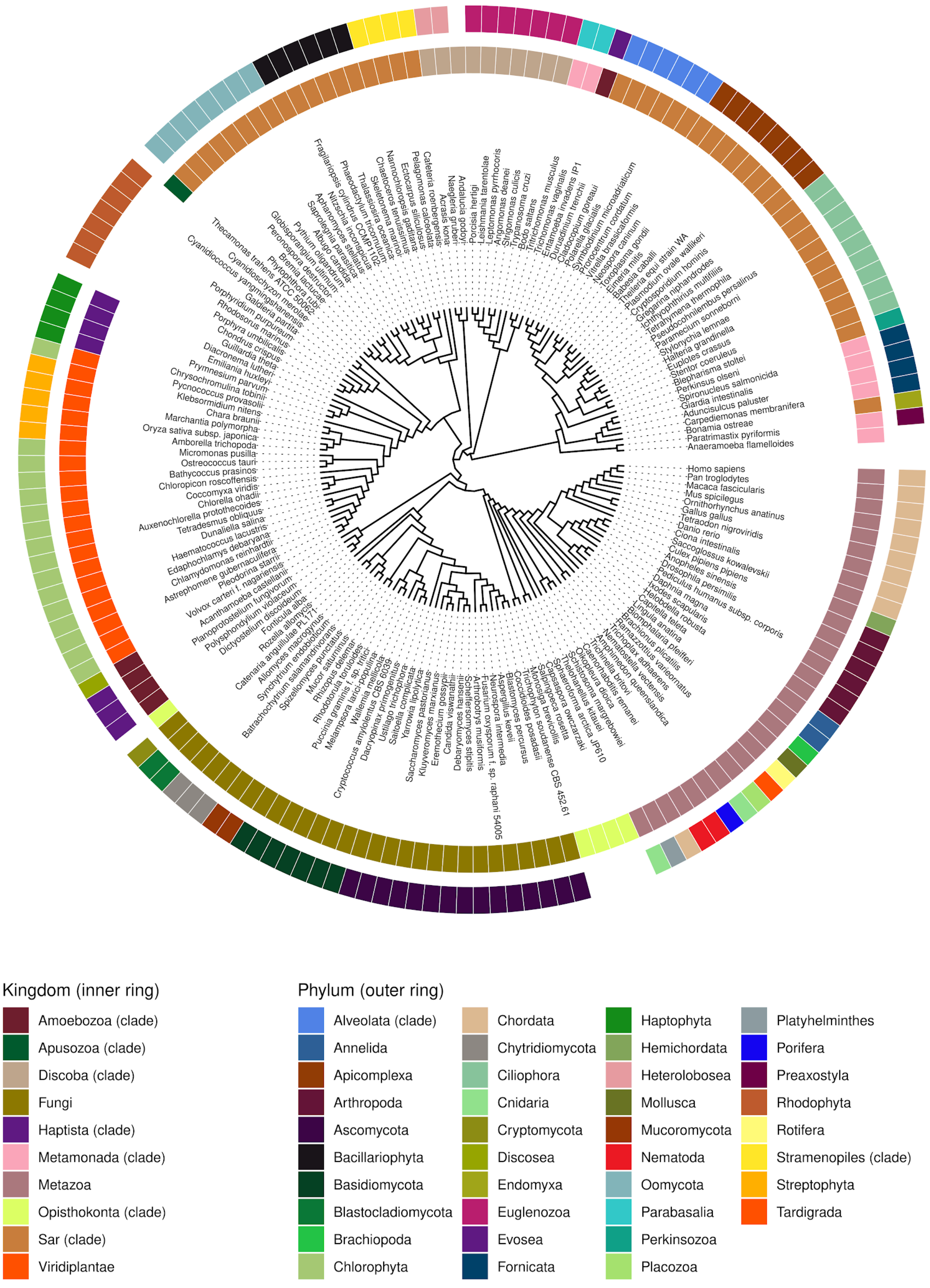

Kingdom (inner ring)
Amoebozoa (clade)
Apusozoa (clade)
Discoba (clade)
Fungi
Haptista (clade)
Metamonada (clade)
Metazoa
Opisthokonta (clade)
Sar (clade)
Viridiplantae
Phylum (outer ring)
Alveolata (clade)
Annelida
Apicomplexa
Arthropoda
Ascomycota
Bacillariophyta
Basidiomycota
Blastocladiomycota
Brachiopoda
Chlorophyta
Chordata
Chytridiomycota
Ciliophora
Cnidaria
Cryptomycota
Discosea
Endomyxa
Euglenozoa
Evosea
Fornicata
Haptophyta
Hemichordata
Heterolobosea
Mollusca
Mucoromycota
Nematoda
Oomycota
Parabasalia
Perkinsozoa
Placozoa
Platyhelminthes
Porifera
Preaxostyla
Rhodophyta
Rotifera
Stramenopiles (clade)
Streptophyta
Tardigrada

Supplementary Figure 2. Cladogram of the broad eukaryotic phylogeny inferred by Phyling. This tree was reconstructed using the eukaryota_odb12 marker set. The inner and outer colored rings represent the kingdom- and phylum-level taxonomic assignments for each sample, respectively.

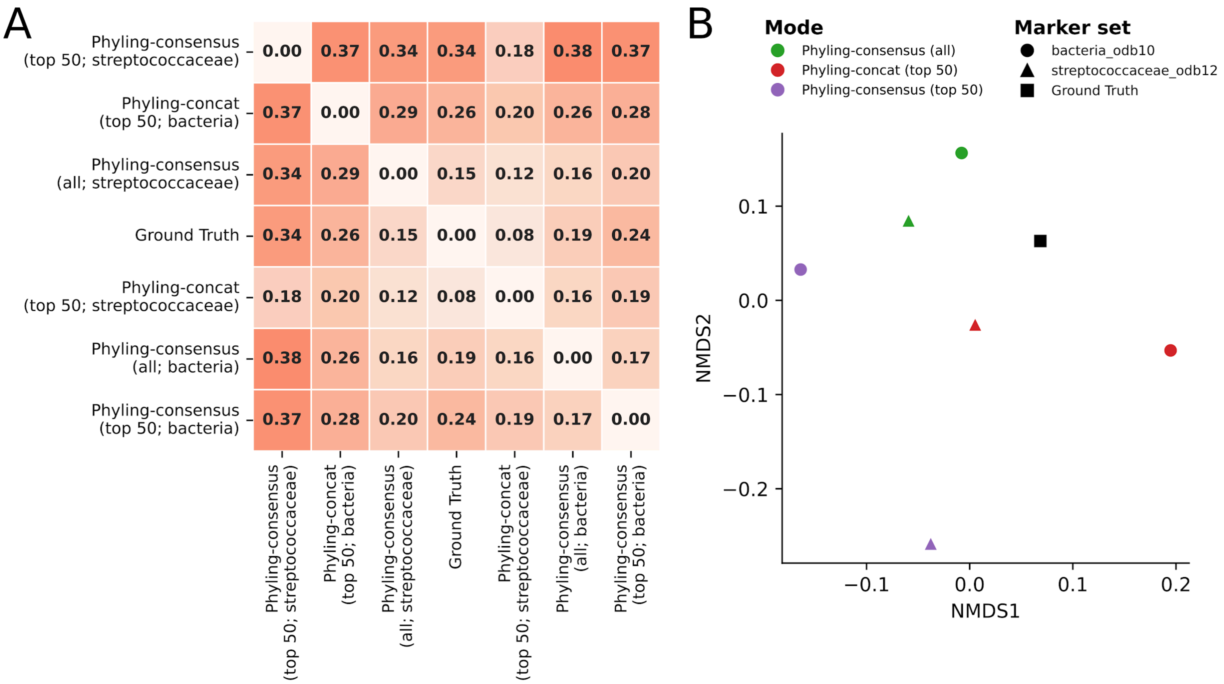

Supplementary Figure 3. Distance among trees inferred by different tools using orthologs identified from the simulated dataset with different marker sets. (A) Generalized RF distance matrix, hierarchically clustered with values ranging from 0 to 1. (B) Corresponding NMDS plot. Colors represent inference methods, shapes denote marker sets used, and the black square indicates the ground-truth tree. All inferences were performed using protein-coding nucleotide sequences.

Supplementary Files

Supplementary File 1. Metadata and repository sources for benchmarking datasets. This file contains five spreadsheets documenting the bacterial, fungal, and broad eukaryotic datasets used in this study. Comprehensive metadata and source information are provided for each sample; corresponding peptide sequences can be retrieved via the download links specified in the “Url” column of each sheet.
